# The Modular Optical Underwater Survey System

**DOI:** 10.3390/s17102309

**Published:** 2017-10-11

**Authors:** Ruhul Amin, Benjamin L. Richards, William F. X. E. Misa, Jeremy C. Taylor, Dianna R. Miller, Audrey K. Rollo, Christopher Demarke, Hanumant Singh, Grace C. Young, Jeremy Childress, Justin E. Ossolinski, Russell T. Reardon, Kyle H. Koyanagi

**Affiliations:** 1 NOAA Pacific Islands Fisheries Science Center, Honolulu, HI 96818, USA; benjamin.richards@noaa.gov; 2 Lynker Technologies LLC, Leesburg, VA 20175, USA; wmisa@lynkertech.com; 3 Joint Institute for Marine and Atmospheric Research, University of Hawaii, Honolulu, HI 96822, USA; jeremy.taylor@noaa.gov (J.C.T.); dianna.miller@noaa.gov (D.R.M.); audrey.rollo@noaa.gov (A.K.R.); christopher.demarke@noaa.gov (C.D.); justin.ossolinski@noaa.gov (J.E.O.); russell.reardon@noaa.gov (R.T.R.); kyle.koyanagi@noaa.gov (K.H.K.); 4 Department of Engineering Sciences, University of Oxford, Oxford OX1 2JD, UK; grace@robots.ox.ac.uk; 5 SeaBed Technologies Inc., Falmouth, MA 02540, USA; seabedtech@gmail.com; 6 The Sexton Corporation, Salem, OR 97302, USA; jeremy@thesextonco.com

**Keywords:** MOUSS, bottomfish, optics, stock assessment, digital stereo-video

## Abstract

The Pacific Islands Fisheries Science Center deploys the Modular Optical Underwater Survey System (MOUSS) to estimate the species-specific, size-structured abundance of commercially-important fish species in Hawaii and the Pacific Islands. The MOUSS is an autonomous stereo-video camera system designed for the in situ visual sampling of fish assemblages. This system is rated to 500 m and its low-light, stereo-video cameras enable identification, counting, and sizing of individuals at a range of 0.5–10 m. The modular nature of MOUSS allows for the efficient and cost-effective use of various imaging sensors, power systems, and deployment platforms. The MOUSS is in use for surveys in Hawaii, the Gulf of Mexico, and Southern California. In Hawaiian waters, the system can effectively identify individuals to a depth of 250 m using only ambient light. In this paper, we describe the MOUSS’s application in fisheries research, including the design, calibration, analysis techniques, and deployment mechanism.

## 1. Introduction

Directly monitoring fish populations through fishery-independent methods, rather than by means of commercial catch data, can provide more accurate and less biased means of assessing the status and trends of fishery stocks to inform stock assessments and resource management decisions. Fishery-independent survey designs are less affected by factors such as market demand, fuel price, and technological advances in fishing methods, all of which can influence commercial effort and catch data, but which may be unrelated to stock status. 

Underwater camera systems have a long history in fisheries research, and the past decade has seen rapid and significant advances in the application of in situ camera technologies to overcome limitations inherent in other survey methods [1]. These systems can be used to generate species metrics including abundance and size, spatial and temporal trends, behavior, and habitat use. Underwater camera systems provide a non-extractive, fishery-independent method for surveying target fish species in their habitat without many of the limitations inherent in traditional survey methods [2]. Species are identified to the lowest possible taxon, counted, and measured following video collection, reducing the logistical constraints and costs of hosting taxonomic experts in the field. Multiple specialists can review and score video footage, reducing inter-observer error, facilitating the transfer of knowledge between analysts, and allowing for the auditing of anomalous records. Video data is archived for future analyses and public education and outreach materials. Furthermore, by broadening the survey range of target fish stocks and increasing sampling capabilities, underwater cameras can supply data that aid in the improvement of fish abundance estimates critical to fisheries management.

The National Oceanic and Atmospheric Administration’s (NOAA) Pacific Islands Fisheries Science Center (PIFSC) has used underwater stereo-camera systems to study abundance and identify juvenile habitat for deep-water snappers [3,4] and to generate species-specific, size-structured abundance estimates of the commercially-important “Deep-7” bottomfish stock [5]. The target “Deep-7” species are Crimson Jobfish (*Pristipomoides filamentosus*), Lavender Jobfish (*Pristipomoides sieboldii*), Oblique-banded Snapper (*Pristipomoides zonatus*), Deep-water Red Snapper (*Etelis carbunculus*), Deep-water Long-tail Red Snapper (*Etelis coruscans*), Rusty Jobfish (*Aphareus rutilans*), and Hawaiian Grouper (*Hyporthodus quernus*). These bottomfish exhibit low rates of natural mortality and a susceptibility to overfishing [6], making non-extractive surveying methodologies particularly ideal in marine protected areas. From 2006 to 2015, camera surveys of the Deep-7 were conducted using a low-light, analogue stereo-camera system called the Bottom Camera Bait Station or BotCam [7]. While this system was versatile and has supported a multitude of studies [5,7,8,9,10,11,12], it was heavy and deployment required a small crane, A-frame, or similar. In addition, it contained ageing components, had limited deployment configurations, and produced analogue, interlaced video that was difficult to annotate. PIFSC redeveloped the BotCam system in 2012 to create MOUSS, the Modular Optical Underwater Survey System. The MOUSS is designed to retain the existing low-light survey capabilities of the BotCam while (a) transitioning to fully digital camera and recording systems that improve image quality, (b) deploying modular components capable of multiple configurations, (c) having smaller dimensions and weighing less than existing systems, and (d) being capable of deployment by vessels and technicians with minimal equipment and training.

## 2. The Modular Optical Underwater Survey System (MOUSS)

### 2.1. Components and Settings

**Figure 1:**
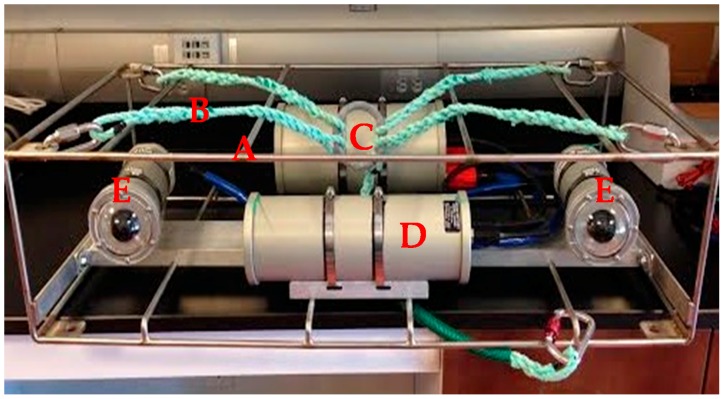
A Modular Optical Underwater Survey System (MOUSS) unit showing (**A**) frame; (**B**) harness; (**C**) digital video recorder (DVR); (**D**) battery module; and (**E**) two camera modules.

The MOUSS consists of two low-light camera modules, a digital recording module, a battery module, and a frame (Figure 1, Table 1). In order to assess the relative performance of the MOUSS, paired MOUSS-BotCam system tests were conducted in 2015 [13]. Preliminary results showed that the MOUSS successfully provided high-quality recordings that matched or exceeded BotCam resolution and image quality [13]. However, the MOUSS low-light sensitivity was slightly lower than the BotCam [13]. The fields of view of the systems were comparable (diagonal angle of view: MOUSS 82° and BotCam 80°), allowing continuity of data streams between the two systems. The BotCam weighs about 49 kg with a 1.50 × 0.75 × 1.00 m frame while the smaller MOUSS weighs 29.43 kg with a 0.25 × 0.50 × 0.75 m frame. The BotCam’s analogue video cameras capture 30 frames per second while the MOUSS’ digital still cameras can capture up to 40 pictures per second. Higher frame rates produce smoother video and improve the chance of capturing fish in orientations ideal for accurately measuring their size. However, high frame rates can also increase costs due to download/processing time and storage space. 

The MOUSS is designed to float approximately four meters above the seafloor, orient down-current, and record images at a downward angle of 15°. This configuration was chosen to match the behavior of target bottomfish species known to school in the water column several meters above the bottom near steep, rocky slopes [6,14]. However, the downward angle and the MOUSS floating distance from the seafloor can change depending on the current. The system was also designed with several charge-coupled device (CCD) sensors including monochrome and color versions that are available in the same physical dimensions, and therefore can be used as interchangeable modules.

#### 2.1.1. Camera Module

**Figure 2:**
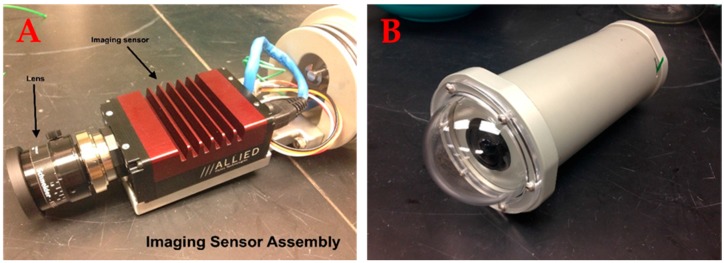
(**A**) The ST-CAM-1920HD camera and (**B**) underwater housing.

Each camera module contains a ST-CAM-1920HD camera (Allied Vision Prosilica GT 1920, Stadtroda, Germany) fitted with 4.8 mm F/1.8 lens (Schneider 21017528, Rueil-Malmaison, France) (Figure 2). Each camera contains a monochrome or colored progressive CCD capable of 2.82 Megapixel resolutions at 1936 × 1456 pixels (Table 1). Frame rate is variable, from zero to 40 frames per second, depending on bandwidth of the recording module (DVR). The DVR has been tested at rates of up to 24 fps. Currently, 12 fps is the standard rate in use at PIFSC. Each camera is enclosed within a 500 m depth-rated underwater housing (Figure 2). Each housing is fitted with a 7.62 cm optically correct polycarbonate dome port, providing an 82° diagonal field of view. Each camera housing is fitted with a single female SubConn^®^ 13-pin PoE (power over ethernet) bulkhead connector to the DVR as well as a pressure relief valve. The camera housings are mounted to a rigid aluminum basebar with a baseline separation of 75 cm and each camera converged at an angle of 5°. Each camera/housing combination has an outer diameter of 8.89 cm and a length of 20.32 cm, weighs 2.32 kg in air, and has a power requirement of 5 W at 7–25 VDC (Volts of Direct Current). 

#### 2.1.2. Digital Video Recording Module (DVR)

**Figure 3:**
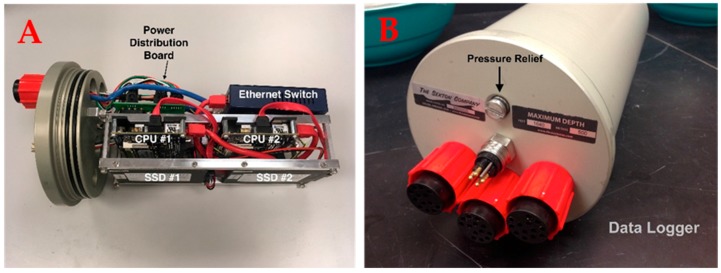
(**A**) The ST-DVR-2HD Digital video recorder (DVR) and (**B**) underwater housing, showing central processing units (CPUs) and solid state hard drives (SSDs).

The ST-DVR-2HD digital video recorder (DVR) (Figure 3, Table 1) consists of an Ethernet switch, power distribution board, two Kontron Pico-ITX-SP 1.6 GHz Intel Atom CPU (central processing units—each dedicated to one camera), and two 512-GB solid state hard drives (SSD) (each dedicated to a camera), all enclosed in a 500-m depth-rated underwater housing (Figure 3). One camera serves as the master (trigger) and the other serves as the slave to maintain synchrony. Each DVR/housing combination has an outer diameter of 15.87 cm and a length of 33.02 cm, weighs 8.16 kg in air, and has a power requirement of 16 W at 9–36 VDC. The DVR housing is fitted with three female SubConn^®^ 13-pin PoE bulkhead connectors, a single male SubConn^®^ 4-pin connector, and a pressure relief valve. The two outer 13-pin connectors are for the cameras while the center 13-pin connector is used for data download. The 4-pin connector is for battery or other power sources. As SSD technology and storage capacity improves, the existing SSDs can easily be replaced with larger and faster modules. Thus, MOUSS is not limited by its data storage capacity. With the current Silicon Graphics Image (SGI) 8 bits format, data can be collected for days without downloading. Data can be downloaded through an Ethernet cable; however, that can take many hours depending on the Ethernet speed and the amount of data. It is currently faster to extract the hard drive to transfer data. It takes two minutes to replace the hard drive; however, the DVR must be opened in a dry space, which can be limited on a small boat. 

#### 2.1.3. Battery Module

**Figure 4:**
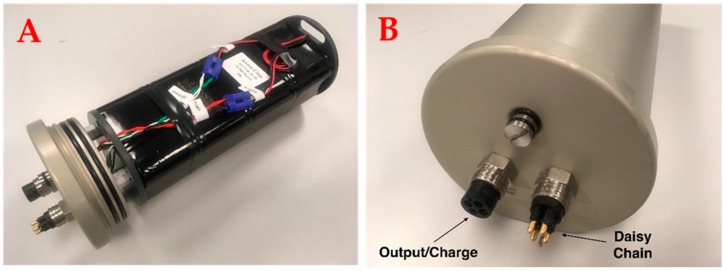
(**A**) The MOUSS 14.4 V, 16 Ah, 16 cell, nickel-metal hydride (NiMH) battery pack and (**B**) underwater housing. Each battery pack can power the MOUSS for up to 6 h. Multiple battery packs can be connected for longer deployments.

The MOUSS is powered by a 14.4 V, 16 amp hour external 16 cell nickel metal hydride (NiMH) battery pack (Figure 4). NiMH was chosen so that the MOUSS units can be shipped throughout the country using standard shipping methods. However, in the near future, lithium ion batteries will be used for the MOUSS units that are not shipped often. The battery is enclosed in a 33.02 × 15.87 cm, 500 m depth-rated underwater housing fitted with a pressure relief valve and one male and one female SubConn^®^ bulkhead connector. The female connector connects to the DVR and is used for charging while the male connector can be used to daisy-chain multiple battery packs together for extended deployments. A single battery pack can power the MOUSS for approximately six hours. As battery technology improves, higher capacity and lighter weight modules can easily replace the existing components.

#### 2.1.4. Frame Module

All of the MOUSS components are mounted within a stainless-steel frame for protection from the surrounding environment and maneuverability during deployment. The two MOUSS camera heads are mounted on a rigid base bar fabricated from a 2.54 × 10.16 cm aluminum “C” channel that is 100.66 cm long. Each camera is mounted using a mounting bracket fabricated from a 2.54 × 5.08 cm aluminum “C” channel that is 15.24 cm long. The baseline separation between the cameras is 75 cm and each camera is converged at an angle of 5°. The basebar is the main structural component, and is typically mounted within a 101.6 × 42.26 × 24.13 cm stainless steel protective cage, but it can be attached to a variety of deployment platforms. Additional sensor modules can also be incorporated within the existing frame. Additional frame components, including tri- or quad-pod legs, can be easily bolted to the existing frame using the pre-drilled mounting holes in the top and bottom corner braces. An optional bait arm can be mounted on the front of the MOUSS frame for baited deployments.

### 2.2. Calibration

**Figure 5:**
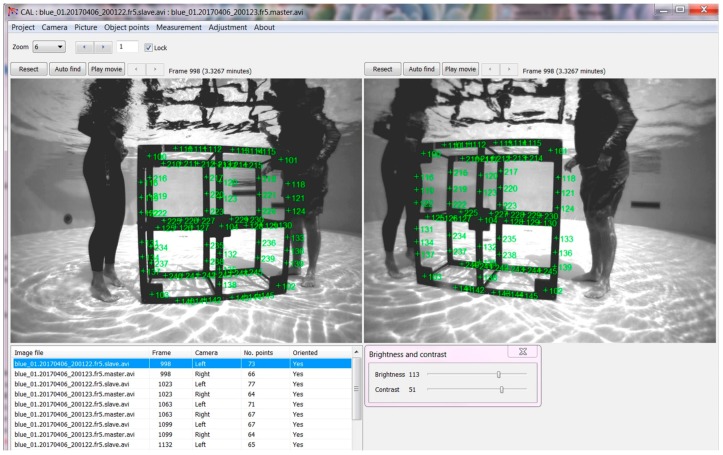
Underwater 3-D “calibration cube” from left and right cameras.

To obtain accurate fish measurements, each pair of the MOUSS cameras is precisely calibrated to the frame on which they are mounted. The calibration process produces calibration files specific to each MOUSS frame, which are used in annotation of video corresponding to that frame. Two of the most common methods for stereo-camera calibration include (a) the use of a two-dimensional checkerboard pattern [15] or (b) a purpose-built three-dimensional calibration cube [16]. The authors of [17] reviewed these techniques and concluded that measurements made with a 3D cube displayed improved accuracy and precision. Currently, PIFSC has standardized this method. The PIFSC calibration process utilizes a 1.0 × 1.0 × 0.5 m custom-made underwater 3-D calibration “cube” (SeaGIS Pty. Ltd., Victoria, Australia), with 77 white dots or “points” of precisely-known spacing (Figure 5). The MOUSS and the cube are placed in a tank of seawater deep enough to completely cover the cube. The cube is centered approximately 1.5 m in front of the paired MOUSS cameras, so that the cube fills most of the field of view. Two people stand in the tank alongside the cube (Figure 5) move the cube into five different orientations: forward-facing; tilted forward; tilted backward; angled to the left; and angled to the right. At each orientation, the cube is held motionless for three seconds to ensure clear imagery. Care is taken to avoid obscuring any of the points with the hands or body, as this may reduce accuracy of the calibration. Following completion of five orientations, the cube is rotated 90° and the same five orientations are repeated in sequence. This continues until five orientations for each of the four sides have been completed, producing a total of 20 cube orientations recorded for each camera pair. A striped mark on the cube’s corner indicates the starting position, ensuring that all five cube rotations are made. 

PIFSC makes use of the SeaGIS CAL software, version 2.0.2 (SeaGIS Pty. Ltd., Victoria, Australia), which provides a photogrammetric bundle adjustment incorporating stereo constraints to produce paired camera calibration files. Paired calibration videos are synced and paused on a frame in which the points appear clearly visible. Each of the calibration cube’s four corner points are marked using a centroid function. The software automatically populates the locations of the remaining points (Figure 5). Depending on orientation, some points will naturally be obscured by the calibration cube itself. Point processing is repeated for each of the 20 paired calibration cube orientations to produce a total of 40 point files. The exact distance between each of the points is precisely known, and the software compares these to the measured distances from the point files, thereby calculating a bundle adjustment and producing a set of calibration files for the camera pair. The bundle adjustment also yields the calibration’s precision with a relative precision of at least 1:5000 required for an acceptable calibration. If the precision value is not within the acceptable level, the point selection process may be partially or entirely repeated to improve “bad” points until an acceptable precision is reached. Finally, measurement accuracy is calculated using SeaGIS EventMeasure^TM^ software, version 3.42 (SeaGIS Pty. Ltd., Victoria, Australia). The calibration videos and corresponding camera calibration files are used to measure distances between a set of points, which are then compared to the known lengths. If all measured lengths are accurate within 2 mm, the calibration is considered acceptable and the camera calibration files are accepted for video annotation.

Calibration is performed both before and after each research mission to ensure that jostling during transport, deployment, and recovery has not altered the orientation of the cameras, invalidating the pre-mission calibration. If a camera is removed, swapped, or adjusted, the pre-mission camera files must be used to annotate all videos recorded before the camera change occurred for a given frame and camera pair, and the post-mission files must be used to annotate all videos recorded after the change occurred. This ensures accurate measurements are obtained at all times.

### 2.3. Deployment

**Figure 6:**
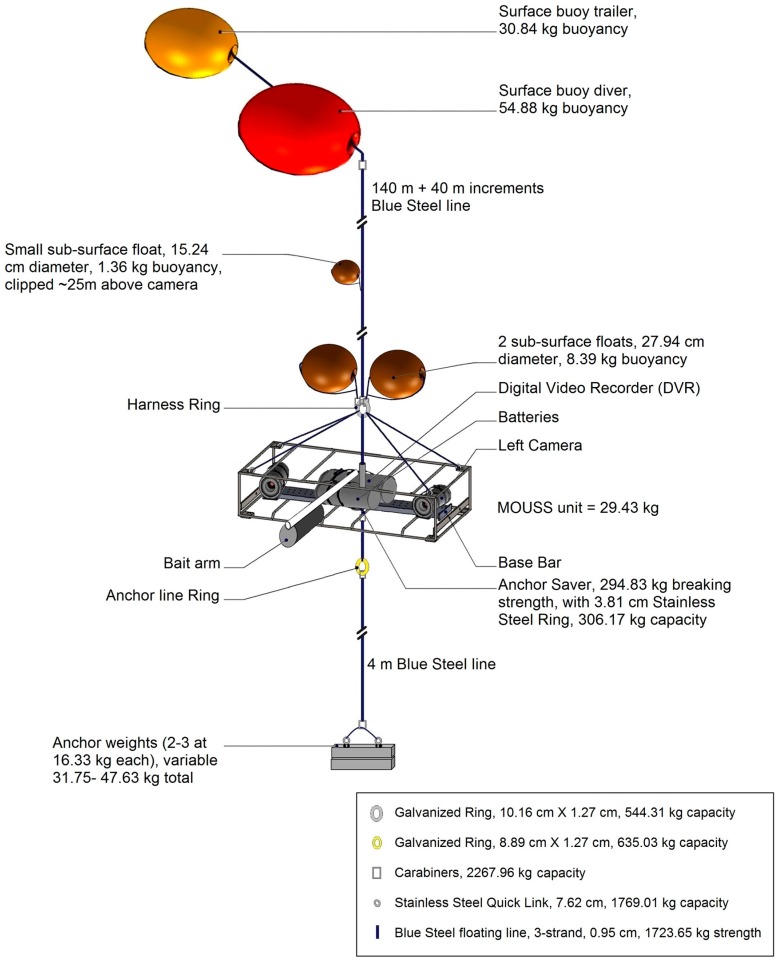
The MOUSS configuration when deployed: MOUSS unit with stereo-video camera system (two camera modules, one DVR module, one battery module, and power cables), two sub-surface floats, bait arm with cage, surface line with two surface buoys, weight, and bottom line with weak link.

PIFSC deploys the MOUSS as an autonomous, stationary video lander from a 5.79 m small survey vessel. The complete system consists of the MOUSS unit with a stereo-video camera system (two camera modules, one DVR module, one battery module, and power cables), two sub-surface floats, a bait arm with cage, a surface line with two surface buoys, an anchor weight, and a bottom line with a weak link (Figure 6). The buoyant force of the surface buoys and the weight of the anchor are distributed solely along the surface line, which runs upward from the anchor, through the center of the MOUSS frame, to the buoys at the surface. This ensures minimal distortion of the MOUSS frame, regardless of load. The MOUSS frame is horizontally tethered four meters above the bottom by means of two sub-surface buoys attached to the upper bridle, which also serves to stabilize the system and to orient the cameras in a 15°-forward down-angle for improved fish detection. The two sub-surface floats are attached above the system giving enough positive floatation to counteract the weight of the MOUSS unit, therefore making it effectively neutrally buoyant. The system is deployed such that the anchor weight is the only point of contact with the seafloor and the cameras float above the bottom. The optional bait arm acts as a weathervane, orienting the field of view of the cameras down current. Should the anchor weight become entrapped, a weak link on the bottom line between the anchor weight and the MOUSS unit is designed to break, allowing MOUSS recovery. PIFSC deploys the MOUSS with a 15-min soak time, determined to be optimal for surveying the Deep-7 bottomfish [8]. For recovery, the surface buoys are grappled using a boat hook and the surface line is recovered using an electric pinch puller, lifting the MOUSS into a davit. The anchor weight is secured, the MOUSS is brought onboard, and the anchor weight is recovered using the pinch puller. 

### 2.4. Data Analyses 

**Figure 7:**
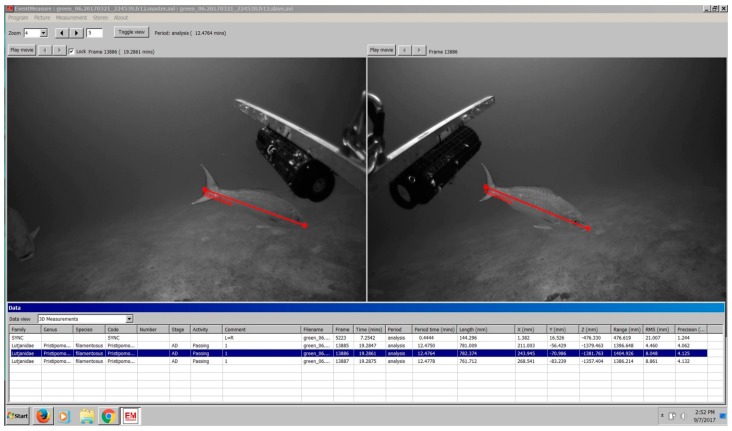
*Pristipomoides filamentosus*, identified and measured using EventMeasure—a screen grab example from the SeaGIS EventMeasure^TM^ desktop software package showing measurement of individual fish.

PIFSC currently uses SeaGIS EventMeasure™ software (Figure 7) for video data analysis to assess relative bottomfish abundance and size composition of the Deep-7 stock. Fish counts are recorded as MaxN [3,7,18,19], the maximum number of individuals of a target species seen at one time during the 15-min analysis period. The MaxN method guards against the recounting of fish during the analysis period, leading to a conservative abundance estimate. Bottomfish fork-length measurements are taken around the time of MaxN to produce species-specific size-structured abundance estimates. PIFSC fish measurement protocols call for five replicate fork-length measurements taken at different video frames for each target individual. Each measurement must have a root mean squared (RMS) error of less than 10 mm and a precision to fork length ratio under 10% to be accepted for analysis [9,10,11]. Furthermore, a between-replicate covariance under 15% is also targeted to ensure that possibly erroneous measurement outliers amongst replicates are avoided. Mean fork length from the five replicate measurements is then used as the representative length for that individual. This is done to increase measurement accuracy. 

**Figure 8:**
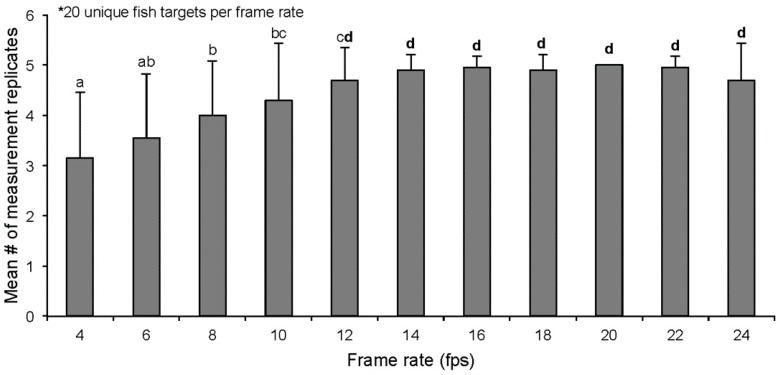
Mean number of *Pristipomoides filamentosus* measurement replicates with standard deviation (SD) from 20 unique fish targets per frame rate (frames per second). Columns that share a letter are not significantly different (PERMANOVA, *p* > 0.05). Error bars indicate +1 SD of the mean.

Assuming that measurement opportunities increase with frame rate, an ideal frame rate is defined as the lowest frame rate able to consistently provide five replicate measurement opportunities for a given target. The MOUSS units with camera frame rates that ranged from 4 to 24 fps, at 2 fps increments, were tested to identify the ideal operating frame rate. Six MOUSS units were set to different frames rates for a sampling day then reset for subsequent days until samples for all 11 frame rates were collected. From recordings of each of the 11 MOUSS frame rates tested, 20 unique individuals of the crimson jobfish (*Pristipomoides filamentosus*) were measured with the goal of taking five replicate measurements per individual aligning with the measurement methodology of previous bottomfish stereo-camera studies [9,10,11]. Measurement targets were selected from the single video frame at which the most *P. filamentosus* were observed in each camera deployment (MaxN) until a dataset of measurement replicate counts for 20 individuals per camera frame rate was achieved. The mean level of measurement replication attainable at each camera frame rate was calculated and a pairwise permutational analysis of variance (PERMANOVA) [20] in Primer 6 (PRIMER-E Ltd., Ivybridge, UK) was used to test for significant differences in measurement replication between frame rates. A single species was chosen for this analysis to lessen possible variability in measurement replicate counts that may be caused by species-specific differences in swimming speed, arrival rate, or behavior. In general, measurement replication for *P. filamentosus* targets increased with higher MOUSS camera frame rates (Figure 8). However, mean levels of attainable measurement replication were not significantly different at frame rates of 12 fps and greater (PERMANOVA, *p* > 0.05; Figure 8). To meet the requirement of five measurement replicates per target individual, a minimum frame rate of 12 fps would be needed.

## 3. Discussion

Stereo imagery has been widely used for the enumeration and measurement of marine organisms [1]. The MOUSS was developed by the NOAA Pacific Islands Fisheries Science Center as a fishery-independent survey system for the Deep-7 stock. The MOUSS has proven to be a viable replacement to the ageing BotCam system. It is capable of collecting fishery data up to a depth of 250 m in Hawaiian waters, uses only ambient light, and provides many key advantages over other similar systems. The MOUSS is modular and highly extensible, allowing for multiple sensor and deployment configurations. In its first two years of use, the MOUSS has been deployed as a stationary camera lander in Hawaii, the Caribbean, the Gulf of Mexico, and off the Californian coast. In Hawaii, the MOUSS has been deployed as a surface-tethered unit with deployment and retrieval of all components, including the anchors. The MOUSS has also been deployed as an untethered unit with an acoustic release used to jettison the anchor weight, allowing the MOUSS to float to the surface for recovery. In the Gulf of Mexico, multiple MOUSS units were outfitted with a lower frame module to rest directly on the substrate. In this case, the units were connected with a ground line, and deployed as a “trap string” with no individual surface tackle. When used off the Californian coast, the MOUSS was outfitted with additional lighting modules for use in low-light, high-turbidity areas. When used in the Gulf of Mexico and off the California coast, the MOUSS was augmented with a Didson^TM^ acoustic imaging sonar module for the comparison of optical and acoustic signatures of target taxa. The digital system has allowed for automated stereo frame matching, eliminating the need for external light syncs and the manual left–right frame matching required by analogue systems. The ability to vary and select the optimal frame rate based on target species allows for the collection of the necessary image data while minimizing file storage resources. The accuracy and precision of length estimates has been improved by the removal of interlacing artifacts generated in analogue systems.

The MOUSS has been adopted by PIFSC as one of two survey gears in a multi-gear, fishery-independent survey for the Deep-7 bottomfish stock. The MOUSS is also being used as the gear against which other sampling technologies are measured in the NOAA Fisheries Untrawlable Habitat Strategic Initiative. Before selecting the MOUSS for the Deep-7 survey, PIFSC evaluated a suite of fishery-independent sampling gears including (a) stationary stereo-video camera platforms (optics), (b) Standardized Cooperative Research Hook and Line fishing (fishing), (c) autonomous underwater vehicle (AUV) stereo-video camera platform (optics), and (d) calibrated active acoustics [5]. 

The calibrated active acoustic system uses sound waves of specific frequencies that bounce off fishes’ swim bladders. This technology can cover a wide range (hundreds of kilometers) of potential fish habitat, and is able to collect data on targets regardless of their position in the water column. While calibrated active acoustics such as EK60 or EK80 can provide reliable estimates of abundance and biomass in many areas [21], PIFSC analysts are, so far, unable to distinguish among Deep-7 species, and between Deep-7 and non-Deep-7 species (potentially resulting in significant false positives in the data), nor provide accurate and precise length information important for stock assessment [5]. Several of the Deep-7 species are closely bottom-associated in highly complex habitats, increasing the likelihood they would be found within the near-bottom acoustic “dead zone” common to these systems, leading to false negatives in the data. Hence, calibrated active acoustics were deemed inappropriate for current Deep-7 operational surveys.

AUVs and towed-camera platforms are capable of carrying the same camera and recording equipment as the BotCam/MOUSS. They can collect video data at multiple depths across a moderate swath (~5 km) of potential habitat and can cover habitat gradients within a single dive. Fishes have shown varying reaction to approaching AUVs [22] and towed camera platforms [23]. As mentioned earlier, Deep-7 species preferentially occupy highly complex hardbottom habitats. Without onboard collision avoidance, these habitats present significant risks to AUVs and towed-camera platforms, increasing the likelihood of equipment loss. Currently, the cost of AUVs, both in terms of equipment and operation, as well as the risk of equipment loss prevents AUVs and towed cameras from efficiently collecting the number of samples required for an operational bottomfish survey in Hawaii.

Traditional survey methods such as hook-and-line fishing can provide a large amount of data with minimal data processing effort, and allow for highly accurate species identification and measurement [5]. Data can be collected from multiple locations within the water column and optimal gear deployment can be determined using acoustic “fish finders.” Having low equipment costs, multiple vessels can conduct surveys simultaneously, allowing for efficient collection of a large amount of data in a short period of time. Research fisheries emulate the methods used by commercial and recreational fisheries. However, these traditional methods have the potential to be biased with respect to size distribution due to the size and type of hooks deployed. Results may also be biased due to the type of bait used, as well as environmental factors that may lead fish to avoid hooks, leading to false negatives in the data. Traditional survey methods are also extractive, and therefore their use is discouraged within protected areas or for targeting overfished stocks, thus limiting their applications.

Stationary stereo-video camera platforms such as the MOUSS have been used by researchers at the PIFSC and University of Hawaii since 1995 [3] and have been used to non-destructively survey Deep-7 bottomfish assemblages associated with the Hawaii bottomfish restricted fishing areas [9,10,11]. Stereo-video camera systems provide a high degree of accuracy and precision with regard to species-specific, size-structured abundance estimates, can easily be deployed and recovered by a team of two to four minimally-trained field staff, and provide standardized data collection regardless of the personnel deploying the system or the deployment location. Their small size and weight means that they can be deployed by hand from a wide range of vessels. Species-specific length-frequency distributions for Deep-7 species have been shown to be comparable between stationary cameras and traditional gears [5]. Interestingly, [5] found that stereo camera landers rarely encountered small, juvenile fishes that would typically be missed by hook-based fishing gears due to size-selectivity. That suggests that juveniles likely prefer different habitats than adults. To avoid unnecessary effects on survey targets, the MOUSS does not typically use artificial lighting. Therefore, unlike with fishing gears or acoustics, the MOUSS is unable to survey depths without adequate ambient light, nor can it survey at night, when certain species are most active and when a significant portion of commercial fishing operations occur. Hence, fishing and the MOUSS were selected as complimentary methods in the PIFSC Deep-7 fishery-independent survey [5]. Methods to extend the MOUSS capabilities beyond its current limits should be considered. Such methods could include, but are not limited to, increasingly sensitive camera sensors, the use of artificial lighting at wavelengths that are undetectable to the target taxa, or acoustic imaging systems such as BlueView^TM^ or Didson^TM^. However, for hydroacoustics or acoustic imaging systems to be informative, it is likely that significant effort would be needed to develop species-specific, size-structured target strength data.

The MOUSS data analysis is conducted using the MaxN method for estimating abundance. While MaxN was developed as a conservative estimator to avoid double-counting individual fishes, abundance estimated by MaxN can be biased upward or downward based on aspects of the assemblage [24]. For species that form patchily distributed schools, MaxN may overestimate abundance as the density of a school seen in a single frame is applied to the overall sample. For species that do not school or exist at low densities, abundance may be underestimated [5]. MaxN also has the potential to bias size estimates. Size measurements made at or near the time of MaxN may be downwardly biased as higher numbers of small individuals can fit within the finite field of view of the camera. For species where smaller size classes are more gregarious, size structure would likewise be downwardly biased. [24] investigated alternative metrics to MaxN and these studies should be continued.

The use of bait to attract target fishes to the camera’s field of view also has the potential to distort the sampling volume, with fishes being drawn from beyond the field of view of the camera. While less of an issue for estimates of relative abundance, uncertainty in the sampling volume presents a significant issue when attempting to generate estimates of absolute abundance. Preliminary comparisons with absolute abundance estimates generated from data collected using fishing gear suggests that the effective sampling area of the MOUSS may extend to a 30 m radius, even though video annotations are restricted to individuals within 7.5 m of the MOUSS.

Finally, annotation and processing time remains an obstacle to the increased use of video-based methods. Translating raw video footage into species-specific, size-structured abundance estimates for stock assessment requires significant effort by highly trained video analysts. The training period for a new analyst can easily take 6 months and can vary among analysts, the number of species in question, and the complexity of the environment. Depending on abundance and species diversity in a given sample, 15 min of stereo-video footage can take from 30 min to 12 h to process, with an average processing time of 2.5 h [5]. In an operational context, it is unlikely that human analysis will be able to keep pace with data collection. Automated image analysis and video processing are active areas of research [25] and development of automated tools for processing optical data streams will be necessary before the data streams can regularly be used for routine assessments. 

## Figures and Tables

**Table 1:** MOUSS Components.

**Camera Module**	
Camera Model	ST-CAM-1920HD (Allied Vision Prosilica GT 1920)
Resolution	1936 × 1456 (2.82 Mpx)
Color/Mono	Color or Mono
Interface	Ethernet IEEE 802.3 1000baseT
Image Sensor	Sony ICX674
Sensor Type (Size)	Progressive CCD (2/3)
Cell Size	4.54 µm
Iris	Fixed
Frame Rate	Variable (0–40 fps) *
Bit Depth	8–14 bits **
Binning	1–8 pixels/rows ***
Gain	0–30 db
Power Requirement	7–25 VDC (5 W)
Lens	Schneider 21017528 4.8 mm, f/1.8
Housing Dimensions	8.89 × 20.32 cm
Weight Including Housing	2.32 kg/Camera
**Digital Video Recording Module**	
Operation System	Linux
Data Storage	2 × 512 GB Solid State Drives
Output	DNG, JPEG, PGM, PNG TIFF, SGI ****
Power Requirement	9–36 VDC (16 W)
Housing Dimensions	33.02 × 15.87 cm
Weight Including Housing	8.16 kg
**Battery Module**	
Type	Nickel-metal hydride (NiMH)
Duration	6+ h
Voltage	14.4 V
Capacity	16 amp hour
Housing Dimensions	33.02 × 15.87 cm
Weight Including Housing	7.48 kg
**Complete System Overview**	
Depth Rating	500 m
Total Weight	29.43 kg
Overall Dimensions (excluding rigging)	46.99 × 21.59 × 102.49 cm

* 12 fps (currently used); ** 8 bits (currently used); *** 2 pixels (currently used); **** SGI-Silicon Graphics Image (currently used), VDC: Volts of Direct Current.
